# Supplementary material for: Chromothripsis during telomere crisis is independent of NHEJ, and consistent with a replicative origin
Source: Genome Res. 2019 May;29(5):737–49. doi: 10.1101/gr.240705.118 (PMC6499312; doi:10.1101/gr.240705.118)
Supplement: Supplemental Material [file supp_gr.240705.118_Supplemental_file_1.zip › contigs/annotated_contigs/DB88/contig.2.DB88_length_706_mean_cov_7.62747875354.docx]

**DB88_length_706_mean_cov_7.62747875354**

```
ATTACACTAAATGCTGTCCAAGTCAATAGATGGCAGAGACTTGGGGACATTGAACCTGTCAGAGAGGTCACAGGCTCCCCAGTTGTGGG
 >chrX:17699129-17699543 + E=2e-237
CAGTCTTCTTTCCAGTTTCCAGAAATGAGACCCTCAAAGAGTGATCTTTAGTAGCCTCATCTTTGTTTATACTTCACTCAACAAGAAGA

CAGGCAAAATAAAGCAAAAAGAACCTTGGAGAGCTCCAGACTTTCCTCTTGTGAATTTGGACCCTGATCCTGGCCTCCCAATCTTCTTT

TGTTTTCAGCCCAGGAAAGGGAACTAATATTTATTAAGCAGTGACACATGCCAAGGATGTTGTTACTTAATTGGAGCCTGAACCACTGC

ATGAAGGCTCTGTGATTCCCCTTTCATAGAAGACTTGCTCTGGATCACACTGATGCTG|GATCACACT|GATGGACGAAAGAGTTTTAA
                                                                    >chrX:17671996-1767
GAAGGCTGGGTGATTTATAATGTCAAATGTCACCGAGGGGTCAGTGGGAGGAGTTCTGCCTGCCAAGGGCAGACCTGGACCCCTAAGGC
2278 - E=1e-153
TTTGGGAAGGCTTGATGGATTGGCTACTGATGGGAGATGTGAAGTGGGTGGGAATACCAGTCAGAGCTTCTCAAAGGTAAAGGGAAGTC

CTTCCCTTAGCCTTGCTTCTCAAAGGACCGGCAGCCCCTGGGGGTTTGGTAGAGATACACCTTCTCAGGCCTTCCCAGACCTGCT
```
